# Incisor position objective for favorable profile in orthodontic camouflage treatment of skeletal class II cases

**DOI:** 10.1186/s40510-025-00582-2

**Published:** 2025-09-30

**Authors:** Yanting Wu, Li Mei, Nanxi Zhu, Ehab A. Abdulghani, Xinlianyi Zhou, Wei Zheng, Yu Li

**Affiliations:** 1 State Key Laboratory of Oral Diseases, National Center for Stomatology， National Clinical Research Center for Oral Diseases, Department of Orthodontics, West China Hospital of Stomatology, Sichuan University, Chengdu, China; 2 Discipline of Orthodontics, Department of Oral Sciences, Faculty of Dentistry, University of Otago, Dunedin, New Zealand; 3 Department of Orthodontics, Faculty of Dentistry, Thamar University, Thamar City,, Yemen; 4 State Key Laboratory of Oral Diseases, National Center for Stomatology, National Clinical Research Center for Oral Diseases, Department of Oral and Maxillofacial Surgery, West China Hospital of Stomatology, Sichuan University, Chengdu, China

**Keywords:** Skeletal class II, Profile, Incisor position objective, Extraction orthodontics, Clear aligner therapy

## Abstract

**Background:**

This study aimed to screen favorable and unfavorable profile outcomes in orthodontic camouflage treatment of skeletal Class II cases, based on which to identify the reference line and the associated value for the optimal incisor position objective (IPO) in such cases.

**Methods:**

A total of 140 Chinese adult skeletal Class II cases were included, who finished orthodontic camouflage treatment with anterior retraction following premolars extraction. Post-treatment lateral cephalograms were trimmed and converted into silhouettes, rated by a panel of orthodontists. The top 30% and bottom 30% ranked cases were included as the favorable and unfavorable profile group respectively. The distances of U1 anterior to the GALL line (U1-GALL), point A vertical (U1-Av), and ANS-Pog line (U1-ANPo) were measured as IPO indicators.

**Results:**

U1-ANPo in the favorable profile group was 4.74 ± 1.65 mm, significantly different from that in the unfavorable profile group (6.02 ± 3.61 mm). U1-GALL was −2.68 ± 2.30 mm and −1.12 ± 2.02 mm, and U1-Av was 4.49 ± 3.97 mm and 6.22 ± 4.42 mm, in the favorable and unfavorable profile group respectively, neither showing significant difference. Among three indicators, only U1-ANPo had a significant discriminatory capacity (AUC = 0.74, *P* = 0.007) for differentiating between the favorable and unfavorable profile group.

**Conclusions:**

In skeletal Class II orthodontic camouflage treatment, the relatively favorable post-treatment profiles are associated with the U1 position anterior to the ANS-Pog line. U1-ANPo of around 4.7 mm could be tentatively proposed as a practical IPO reference in treatment planning for such cases.

## Background

Establishing the incisor position objective (IPO) represents a pivotal step in orthodontic treatment planning [1], particularly within the realm of clear aligner therapy (CAT). In CAT, the IPO dictates the terminal position of the digital setup, which determines the extraction sites and anchorage design [2]. IPO is typically set on the pre-treatment cephalogram, comprising three elements: the sagittal location, the vertical location and the labiolingual angulation of the central incisors. Since the incisor retraction results in retrusion of the lips, the sagittal location within IPO is crucial for the post-treatment facial profile [3].

In Downs and Ricketts cephalometric analysis, sagittal IPO of the mandibular central incisor (L1) is indicated as its distance in front of the A-Pog line [4]. In the Tweed triangle, for Caucasian patients with lip protrusion, L1 should be uprighted to get an FMIA angle of 65° for obtaining an esthetic profile [5]. Meanwhile, the sagittal IPO of the maxillary central incisor (U1) has been paid increasing attention to, and it is taken as a determinant of the ideal soft tissue profile [6]. In the McNamara analysis, U1 is suggested to be positioned 4–7 mm in front of the FH vertical line passing point A [7]. According to Six Elements of Orofacial Harmony, the FA point of U1 is supposed to be positioned 0–4 mm anterior to the goal anterior limit line (GALL) [8, 9]. A recent study suggested the position of U1 relative to the perpendicular line passing anterior nasal spine (ANS) as an IPO indicator [8]. Another study established a multivariable prediction equation for the distance from U1 to the nasion-pogonion(N-Pog) line to determine the IPO [10].

**Fig. 1:**
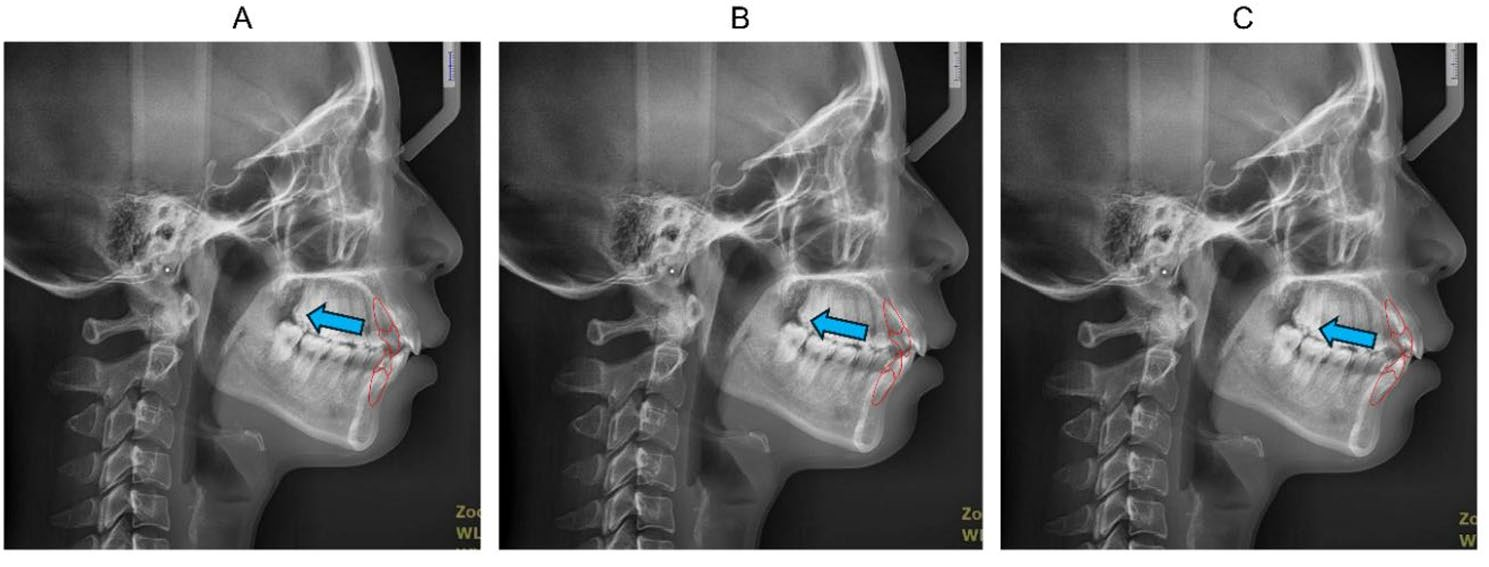
Setting IPO for the treatment planning in CAT: When making treatment plan for a CAT case needing anterior retraction, orthodontists are advised to first set IPO of the maxillary central incisor (U1) on the pre-treatment cephalogram, and then submit the information, the U1 retraction amount for instance, to the technician for defining the terminal position of incisors in the digital set-up. **A**, IPO option #1 (retraction of U1 by 8 mm); **B**, IPO option #2 (retraction of U1 by 6 mm); **C**, IPO option #3 (retraction of U1 by 4 mm)

Skeletal Class II malocclusion, characterized by maxillary protrusion and/or mandibular retrusion, is highly prevalent [11]. For cases of mild to moderate skeletal Class II malocclusion, orthodontic camouflage treatment is a common option [12, 13]. This treatment modality usually involves retraction of the incisors to reduce lip protrusion and improve profile esthetics [14–17]. Therefore, the sagittal or anterior-posterior IPO is particularly important in skeletal Class II camouflage treatment. In treatment planning of a skeletal Class II camouflage case to be managed with CAT, orthodontists are advised to first set the sagittal IPO on the pre-treatment cephalogram (Fig. 1), and then request the technician to define the terminal position of the incisors in the digital set-up based on the determined IPO. Nevertheless, it remains unclear whether there is a rational indicator to delineate the optimal IPO for skeletal Class II camouflage orthodontic treatment.

Hence, this study aimed to collect skeletal Class II cases finished with orthodontic camouflage treatment and screen those with the most favorable and unfavorable profile outcomes, based on which to identify the reference line and the associated value for the optimal IPO of U1. The sagittal position of U1 was to be assessed with three reference lines respectively, including the GALL, the FH vertical line passing point A(Av), and the ANS-Pog line (ANPo).

## Materials and methods

This is a retrospective study, approved by the Ethics Committee of West China Hospital of Stomatology, Sichuan University， China (WCHSIRB-D-2021-402). Chinese adult patients were collected, who received orthodontic treatment in the orthodontics department at West China Hospital of Stomatology, from 2019 to 2023. Sample size calculation was done using the formula method [18], with α set at 0.05 (two-tailed), Z0.05/2 = 1.96, 1-β set at 0.8 (one-tailed), and the calculated minimal sample size is 118. According to the inclusion and exclusion criteria, a total of 140 patients (47 males and 93 females) were included. The average age was 25.2 ± 5.5 years at the initial consultation, and 28.5 ± 6.2 years at the end of treatment, with an average treatment duration of 3.3 ± 1.7 years.

Inclusion criteria : (1) age ≥ 18 years old at the beginning of treatment and ≤ 35 years old at the end of treatment; (2) mild to moderate skeletal Class II malocclusion with 4° ≤ ANB ≤ 7° before the treatment; (3) orthodontic camouflage treatment was carried out with anterior retraction following extraction of bilateral maxillary premolars; (4) with normal post-treatment overjet; and (5) full records were available before and after the treatment. Exclusion criteria: (1) patients with missing or abnormally shaped maxillary or mandibular central incisors; (2) active periodontal diseases; (3) change of the MP-FH angle larger than 2° after the treatment.

**Fig. 2:**
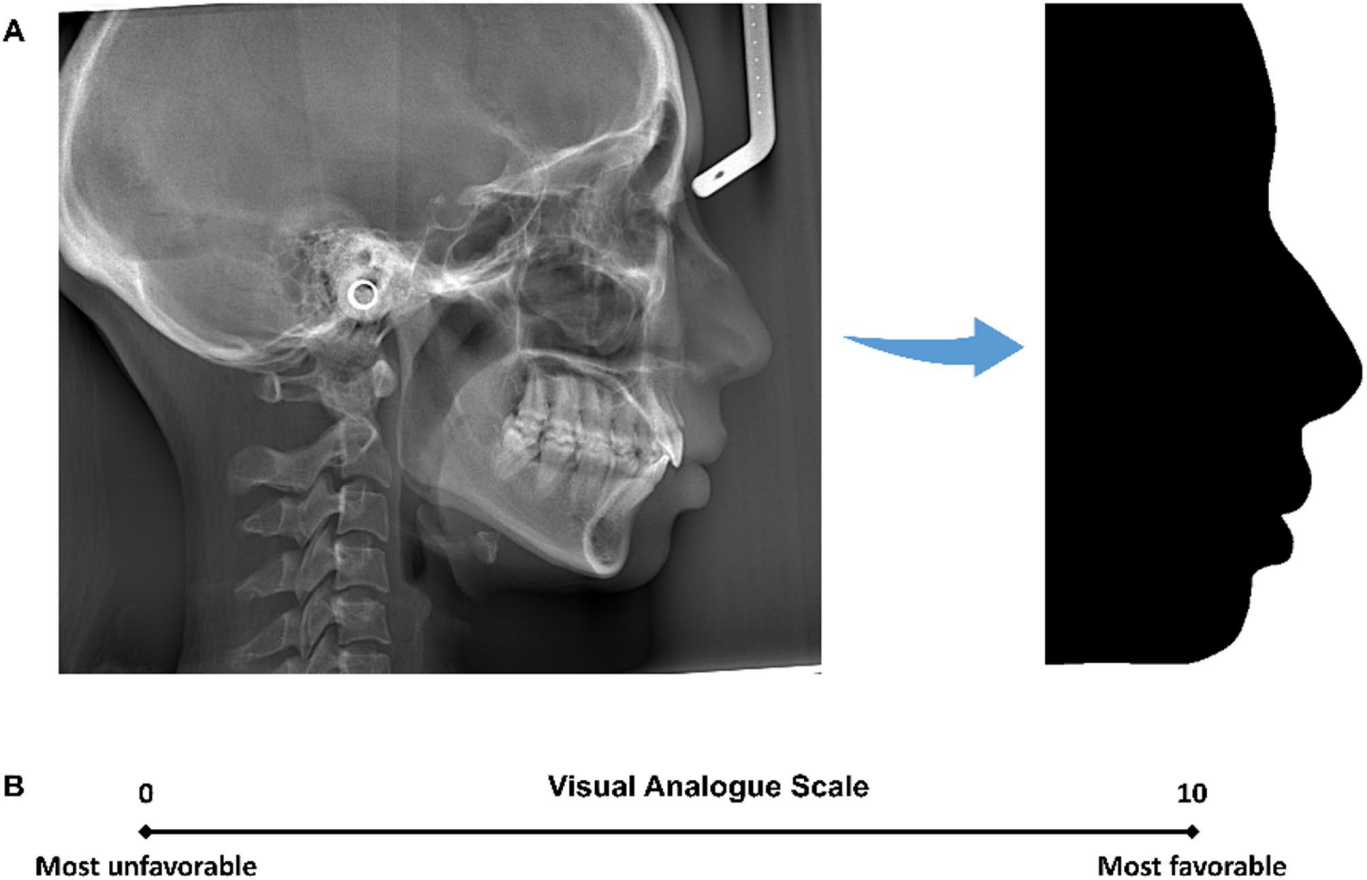
Assessment of the profile attractiveness: **A**, The lateral cephalograms were trimmed and made into “black profile” silhouettes for the panel evaluation; **B**, The visual analogue scale (VAS) approach was used to rate the profile attractiveness

Lateral cephalograms were obtained with the patients’ lips slightly closed and their teeth occluded in maximal intercuspation. The cephalograms were reoriented by superimposing the lateral photographs taken in the natural head position. Notably, the patients’ post-treatment lateral cephalograms were digitally processed using Adobe Photoshop 2020 (Adobe Inc., San Jose, CA, USA). The soft tissue outline of the profile was meticulously traced with the pen tool, and then the outline was filled in black. “Black profile” silhouettes were thereby generated, with all the dental and skeletal information removed (Fig. 2A). As a result, the evaluators could concentrate on the profile outline for esthetic assessment. The male and female patients were not discriminated, since the gender characteristics could not be identified on the silhouettes of cephalograms.

A panel of four orthodontists evaluated the profile silhouette images using a visual analogue scale (VAS) (Fig. 2B) [19], who were blinded from the patients’ other information. After a 4-week interval, the same evaluators re-rated all the images shown in a randomly rearranged order. The intraclass correlation coefficient (ICC) [20] values for the repeated ratings exceeded 0.85 for all the four evaluators, and the ICC value for the inter-rater agreement was 0.83, suggesting good reliability of the evaluation. Additionally, the obtained rating scores showed normal distribution.

**Fig. 3:**
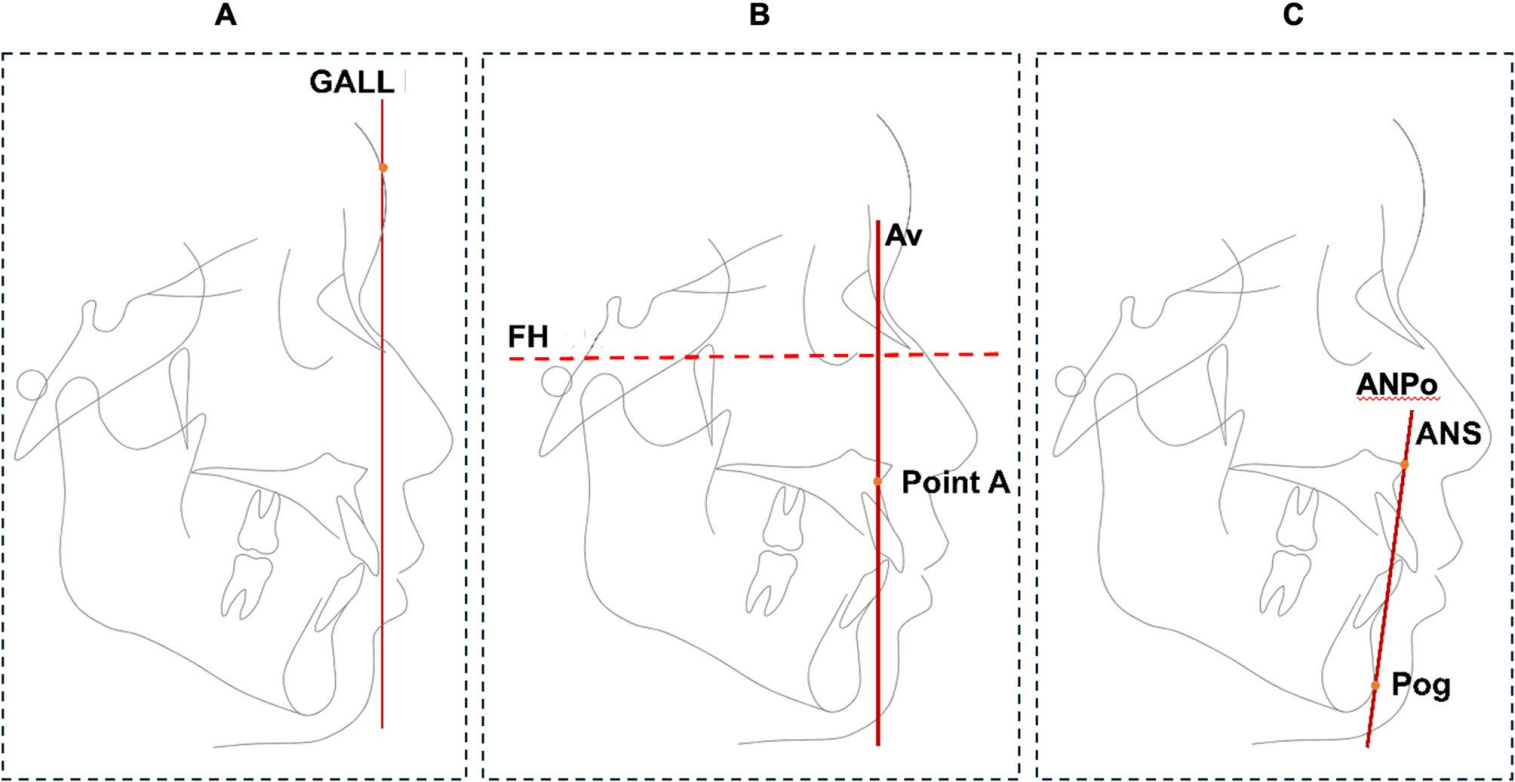
Three IPO reference lines: **A**, GALL: The goal anterior limit line; **B**, Av: The line passing the point A and vertical to the Frankfort Horizontal (FH); **C**, ANPo: The ANS-Pog line. U1-GALL, U1-Av, and U1-ANPo represent the distance of the incisal point of U1 anterior to their respective reference lines

Cephalometric measurements were done on the original radiographs by a single examiner, using the SmartOrtho software (Sichuan University, Chengdu, China), in triplicates with the average outcomes calculated. Three candidate reference lines for IPO were selected, including the GALL [8] the FH vertical line passing point A(Av), and the ANS-Pog line (ANPo). Thus, three IPO indicators were established, namely U1-GALL, U1-Av, and U1-ANPo, representing the distance of the incisal point of U1 anterior to their respective reference lines (Fig. 3). Other than the three IPO indicators, routine cephalometric measurements were also performed.

**Fig. 4:**
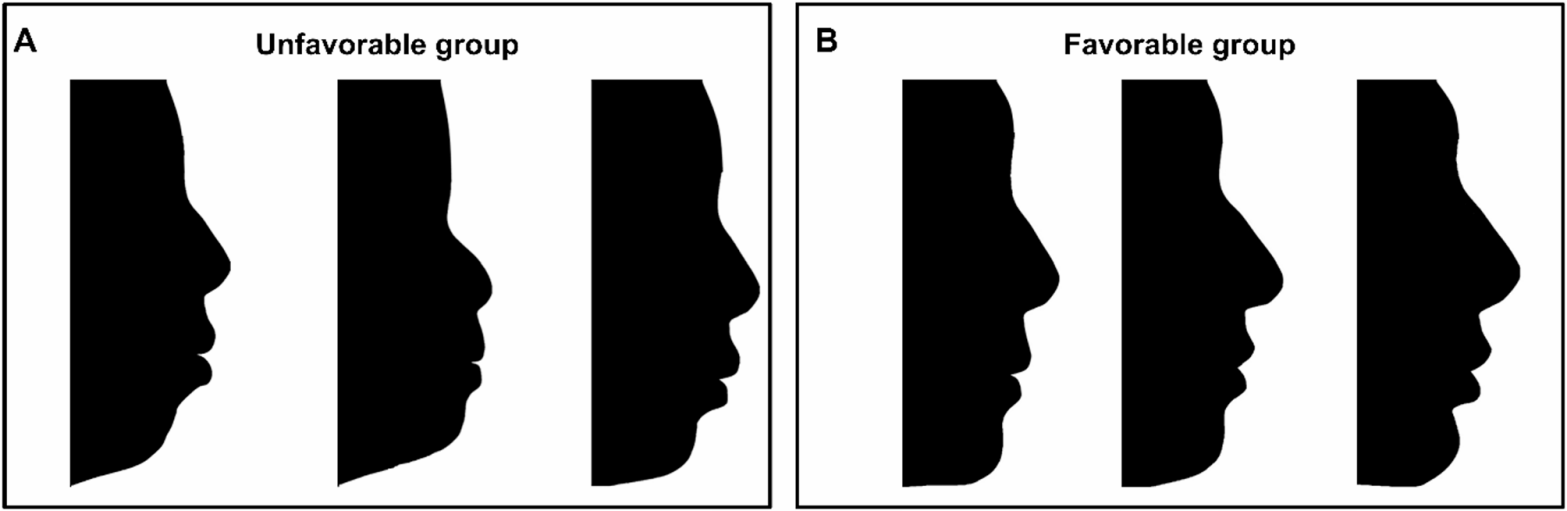
Three representative silhouettes in the post-treatment unfavorable and favorable profile group respectively: **A**, The unfavorable profile group; **B**, The favorable profile group

The statistical analysis was performed using SPSS Statistics Version 25.0 (IBM Corp., Armonk, NY, USA). Firstly, the Pearson correlation test was used to evaluate the association of U1-GALL, U1-Av, and U1-ANPo with the profile ratings. Then, a rank-based grouping method was adopted to enhance discrimination. Based on the rank of the profile ratings, the top 30% and bottom 30% cases were considered as the favorable profile and unfavorable profile groups respectively (Fig. 4). This stratification circumvented ambiguity in the mid-range and revealed more pronounced differences between the two representative groups. The measurements of IPO indicators in the favorable and unfavorable profile groups were calculated and compared using grouped t-test analysis. The normality of distributions was verified using the Shapiro-Wilk test, and homogeneity of variances between groups was tested using the Levene test. Furthermore, receiver operating characteristic (ROC) curve analysis [18] was employed to assess the accuracy of the tests. A ROC curve that is closer to the upper left corner indicates higher test accuracy, while proximity to the diagonal line (y = x) suggests accuracy akin to random guessing. A significant level of *P* < 0.05 was adopted.

## Results

Within the full sample(*n* = 140), the pre-treatment U1-GALL was 1.48 ± 1.13 mm, U1-Av was 8.65 ± 3.47 mm, and U1-ANPo was 8.93 ± 3.80 mm; the post-treatment U1-GALL was − 2.10 ± 2.04 mm, U1-Av was 5.35 ± 2.22 mm, and U1-ANPo was 5.45 ± 2.30 mm. The Pearson correlation tests showed no significant association of the post-treatment U1-ANPo (*r* = − 0.12, *P* = 0.355), U1-GALL (*r* = 0.06, *P* = 0.3764) or U1-Av (*r* = 0.03, *P* = 0.8233) with the full-sample profile esthetic ratings. 

As described above, the ranked top 30% and bottom 30% cases were then designated as the favorable and unfavorable profile groups respectively. The average rating score was 6.38 ± 0.73 in the favorable profile group (*n* = 42), significantly higher than that in the unfavorable profile group (1.84 ± 0.68, *n* = 42). Three representative images in the unfavorable and favorable profile groups were shown in Fig. 4.

**Fig. 5:**
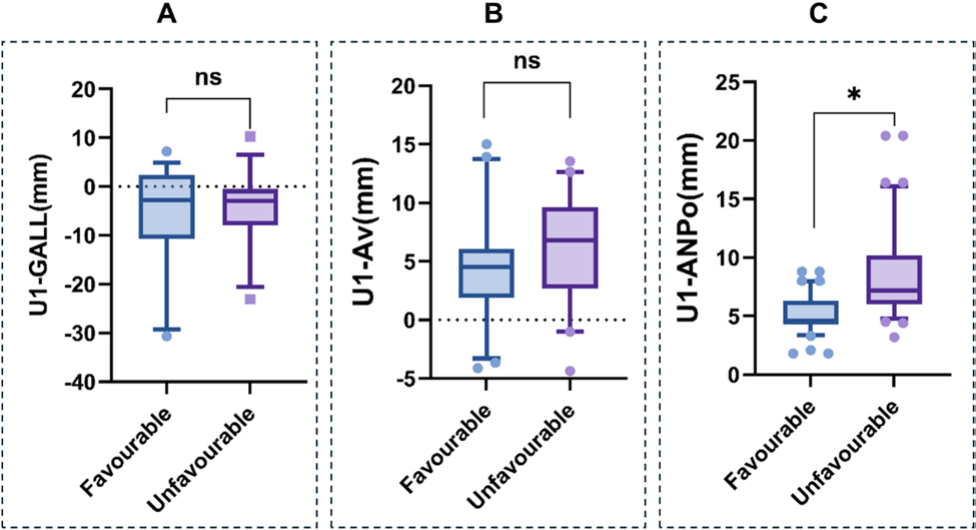
Post-treatment values of the three indicators in the favorable and unfavorable profile groups: **A**, U1-GALL; **B**, U1-Av; **C**, U1-ANPo. Box plots depict the median (central line), interquartile range (boxes), and range excluding outliers (whiskers). Points represent individual observations. * *P* < 0.05, ns: no significant difference, grouped t-test analysis

The values of the three IPO indicators in the favorable and unfavorable profile groups were shown in Table 1. No significant differences were found between the two groups for any pre-treatment indicator. Notably, the post-treatment U1-ANPo in the favorable profile group was 4.74 ± 1.65 mm, significantly (*P* = 0.004) different from that in the unfavorable group (6.02 ± 3.61 mm) (Fig. 5), suggesting U1-ANPo as an effective indicator discriminating profile esthetics. In contrast, the post-treatment U1-GALL was − 2.68 ± 2.30 mm and − 1.12 ± 2.02 mm (*P*= 0.644), and U1-Av was 4.49 ± 3.97 mm and 6.22 ± 4.42mm (*P*= 0.495), in the favorable and unfavorable profile groups respectively, neither showing significant differences between the two groups.

**Table 1:** The pre- and post-treatment U1-GALL, U1-Av and U1-ANPo in the favorable and unfavorable profile groups

Group	Pre-treatment (mm)			Post-treatment(mm)		
	U1-GALL	U1-Av	U1-ANPo	U1-GALL	U1-Av	U1-ANPo*
Favorable profile	1.48 ± 1.13	8.65 ± 3.47	8.93 ± 3.80	-2.68 ± 2.30	4.49 ± 3.97	4.74 ± 1.65
Unfavorable profile	2.68 ± 2.30	6.22 ± 4.42	6.02 ± 3.61	-1.12 ± 2.02	6.22 ± 4.42	6.02 ± 3.61

U1-GALL, U1-Av, and U1-ANPo represent the distance of the incisal point of U1 anterior to their respective reference lines. * *P* < 0.05, compared between the favorable and unfavorable profile groups using grouped t-test analysis.

**Fig. 6:**
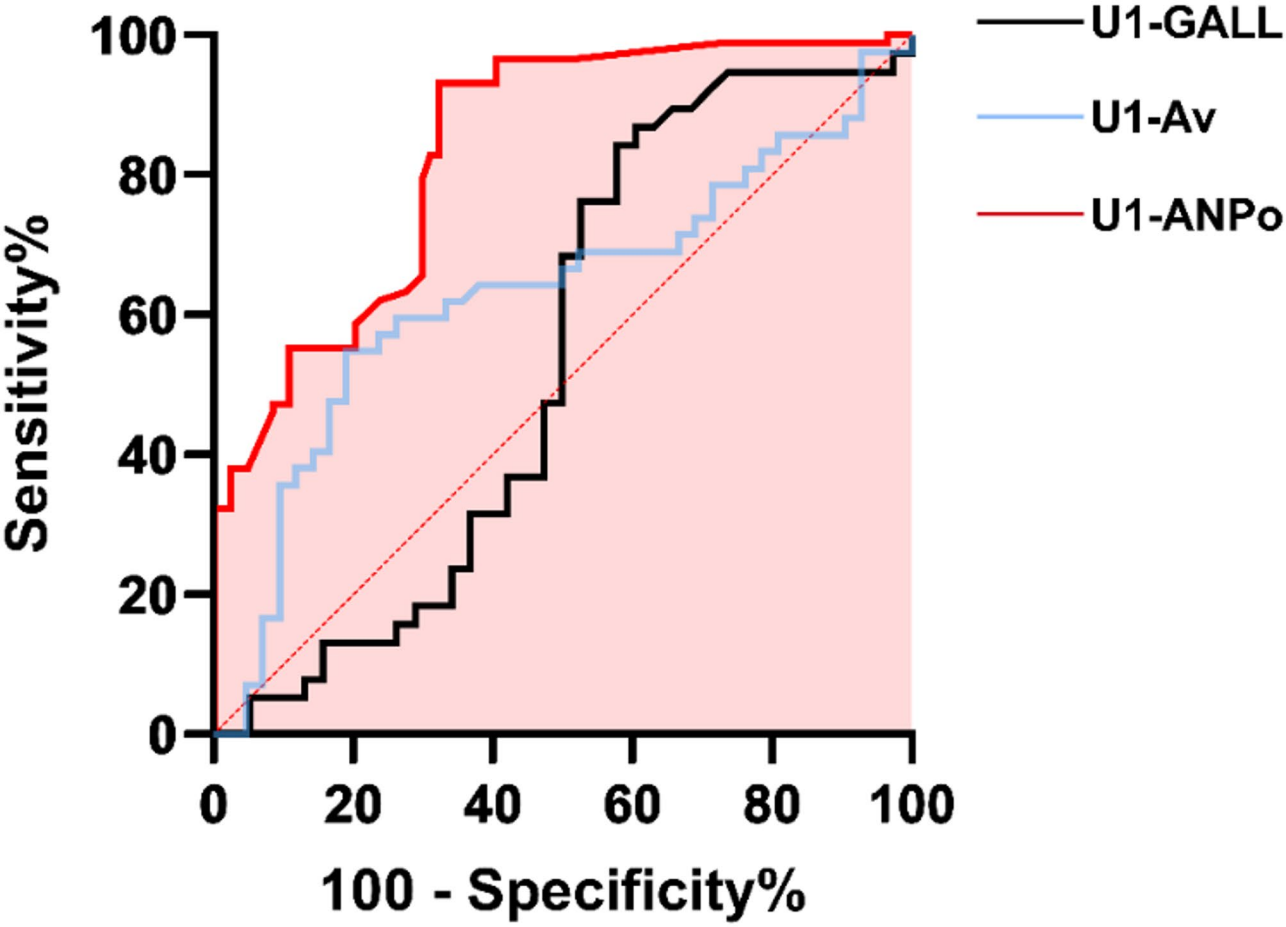
ROC analysis of U1-ANPo (red curve), U1-GALL (black curve), and U1-Av (blue curve) to discriminate between the favorable and unfavorable profile groups. The area under the curve (AUC) represents discriminatory capacity. The red filled portion indicates the AUC of U1-ANPo. The red dotted line indicates that y = x

In the ROC curve analysis, the area under the curve (AUC) of U1-ANPo was 0.74 (*P* = 0.007, Fig. 6), showing a strong discriminatory capacity [14] for the favorable and unfavorable profile groups. The ROC curve for U1-ANPo also showed a greater separation from the line y = x. No insignificant discriminatory performance was found for U1-Av (AUC = 0.63, *P* = 0.392) or U1-GALL (AUC = 0.56, *P* = 0.578).

## Discussion

The IPO is crucial in orthodontic treatment planning, especially for the extraction and anterior retraction cases such as bimaxillary protrusion [21] and camouflage treatment of skeletal Class II [22]. The present study investigated the effectiveness of the GALL, Av, and ANPo as reference lines for sagittal positioning of U1, based on the post-treatment profile esthetics in skeletal Class II camouflage orthodontic treatment. It was found that only the indicator U1-ANPo is associated with the favorable profile outcome, and the optimal U1-ANPo is 4.7 mm, suggesting that U1 positioned about 4.7 mm in front of the ANS-Pog line may hold promise for a favorable profile in orthodontic camouflage treatment of mild to moderate skeletal Class II patients.

Although most IPO-related studies have investigated skeletal Class I malocclusion, a recent study found the U1-GALL to be 2.05 ± 3.67 mm before and − 2.84 ± 3.37 mm after the orthodontic treatment of skeletal Class II cases [21].For an individual skeletal Class II patient, retraction of U1 to an appropriate extent, indicated by decreased U1-GALL, may improve the profile esthetics. Nevertheless, an IPO based on U1-GALL might be unperfect, since the position of the GALL line depends on the patient’s forehead protrusion, which varies among individuals. Of note, the E-line and Z-angle that can effectively predict the profile esthetics [23] are in relation to the lower third face only, whereas not to the forehead. U1-Av has also been used in the IPO determination in skeletal Class I patients [24]. However, the Av line does not involve the position of the chin, which may well be abnormal in the skeletal Class II malocclusion. In contrast, the ANPo line is composed of ANS and Pog, two landmarks representing the sagittal position of the maxilla and mandible respectively, making it potentially more effective as the reference line for incisor positioning in skeletal Class II malocclusion, which encompasses an abnormal maxillomandibular relationship. Hence, U1-ANPo, but not U1-GALL or U1-Av, is associated with the favorable resultant profile in the camouflage treatment of skeletal Class II malocclusion, probably because the reference line (ANS-Pog) comprises both the maxillary and mandibular frontier landmarks.

A similar IPO indicator, U1-APog, has been observed in a previous study [8]. Why has A-Pog not been used as the reference line in the present study? Some studies have shown that retraction of the maxillary incisors, especially their roots, may lead to corresponding retraction of point A due to alveolar bone modeling [25–27], making the A-Pog line changeable with the orthodontic treatment. Compared to point A, point ANS should be more stable during orthodontic treatment with incisor retraction. Therefore, U1-ANPo, instead of U1-APog, has been used as the IPO indicator in the present study.

Images used for profile evaluation are often lateral photographs or cephalograms. In the present study, “black profile” silhouettes of the trimmed lateral cephalograms were utilized. This approach has eliminated influences from the skin color, skin condition, hair shape, and skeletal and dental morphologies in the esthetic evaluation of the profile outcomes. Similar“black profile” silhouettes were generated from lateral photographs rather than cephalograms in another study [28].That approach leaves the hair contour on the silhouette, which may make it look more real, while the hair shape could also be a confounding factor. Additionally, cephalograms are taken in a more standardized way than lateral photographs.

In this study, cases of “mild to moderate skeletal Class II malocclusion” were included based on the criterion “4° ≤ ANB ≤ 7°”. These cases were not further grouped based on the mandibular plane angle (MPA) for the following reasons: Firstly, classic IPO indicators, such as U1-GALL and U1-Av, do not originally incorporate MPA into their conceptual framework. Secondly, the influence of MPA is partially accounted for within the ANB angle, as a high angle is typically associated with a retruded mandible and vice versa. Furthermore, the superior performance of U1-ANPo in the results can be partially attributed to its inclusion of the point Pog in the reference line, and the role of MPA may have been encompassed more or less in this context, as it directly affects the position of Pog.

Although the indicator U1-ANPo reflects the relationship between the incisor, maxilla, and mandible, it does not contain soft tissue, which also plays important roles in the facial profile esthetics. It was found that, for patients of Class I occlusion with thick lips, the position of the incisor may not affect the perception of the profile [29]. Another study showed that the lip thickness may negatively correlate to the ratio of upper lip retraction upon maxillary incisor retraction [30]. A variety of approaches have been attempted to predict lip changes following incisor retraction, including proportional conversion [31] logistic regression model [32, 33], specialized software [34], and artificial intelligence [35]. Proportional conversion assumes that the soft tissue changes are linear to the dental changes, which often does not hold true. Logistic regression models also perform inadequately when dealing with complex nonlinear relationships. Specialized software based on algorithms or artificial intelligence may be advantageous in dealing with this issue; however, its prediction accuracy depends on the quantity and quality of the training data. Additionally, confounding factors, such as the aging-dependent thinning of lips [36], may also affect the accuracy of predicting profile changes after incisor retraction. Therefore, when applying the IPO indicator in treatment planning, individual soft tissue characteristics and the potential influences should also be considered.

Worth mentioning, as all the patients and evaluators in the present study were Chinese, the present findings, especially the optimal value of U1-ANPo for a favorable profile, should not be incautiously extrapolated to other ethnicities due to inherent differences in facial anatomy, soft tissue characteristics, and cultural perceptions of esthetics. Further studies with diverse populations are warranted to testify the applicability of U1-ANPo across races.

## Conclusions

In orthodontic camouflage treatment of skeletal Class II patients, U1-ANPo is associated with post-treatment profile attractiveness. In treatment planning for such cases, a straightforward and pragmatic IPO parameter could be tentatively proposed as U1-ANPo of around 4.7 mm, subject to necessary adjustment based on individual soft tissue features and esthetic perceptions.

## Data Availability

The data underlying this article will be made available at reasonable request to the corresponding author.
